# Voice gender diversity: expression, perception and acoustics

**DOI:** 10.1098/rsos.251193

**Published:** 2025-10-22

**Authors:** Victor Rosi, Carolyn McGettigan

**Affiliations:** ^1^ Department of Speech, Hearing and Phonetic Sciences, University College London, London, UK

**Keywords:** voice gender perception, gender diversity, voice identity, gender identity, speech acoustics, gender expression

## Abstract

The voice is a key marker of gender identity, yet gender non-conforming speakers often diverge from binary vocal norms. This review synthesizes findings from 45 studies published between January 2020 and January 2025 examining how gender is expressed and perceived for the voices of gender-diverse individuals. We report how gender identities and expressions are measured, how gender-diverse voices are perceived, and how acoustic features relate to both self-reported and perceived gender. While binary frameworks still influence the experimental methods used, a shift towards more inclusive models is emerging. Gender-diverse voices are frequently externally perceived or acoustically represented as falling between binary categories or as misaligned with affirmed gender identity. Similar to binary voice gender, fundamental frequency and formants are key components of expressed and perceived gender. However, these findings may be biased owing to the binary understanding of gender in the methods used, a general focus on transfeminine voices, and few investigations on the impact of listeners’ identity. A few studies reported that gender non-conforming listeners tend to have a distinct understanding of voice gender compared with cisgender listeners. To move forward, future research should adopt inclusive perceptual tasks, account for listener demographics, and further explore self-perception of voice gender.

## 1. Introduction

Gender is one of the most salient identity markers conveyed through the human voice [1]. However, for gender non-conforming speakers—such as transgender and non-binary individuals—vocal characteristics may diverge from binary norms, altering both their self-perception of voice gender and how others perceive it. This is particularly true for individuals who have undergone gender-affirming voice training (GAVT) or gender-affirming hormone therapy (GAHT), which can modify vocal cues [2,3]. As a result, there is a growing number of studies investigating the voices of gender non-conforming individuals, exploring both perceived gender and acoustic cues through speech datasets that showcase gender-diverse voice identities.

Over the past decades, the public understanding of gender diversity has expanded, leading to a more nuanced and inclusive terminology for gender identity [4]. Recently, numerous studies have emphasized the importance of using respectful and appropriate methods to assess gender identity in both academic research and society [5–9]. As a result, various questionnaires have been developed to examine gender identity and expression, with some specifically addressing gender dysphoria, i.e. the distress a person undergoes when their gender identity mismatches their gender assigned at birth [10,11]. In voice and speech research, the gender identity of gender-diverse individuals (i.e. individuals whose gender identity does not align with their sex assigned at birth), like transgender or non-binary individuals (i.e. individuals whose gender identity does not fit exclusively within the categories of male or female), has been typically assessed using either categorical response formats or free-text entries. The Transgender Woman Voice Questionnaire (TWVQ) [12]—originally designed for clinical settings in GAVT—has become the most widely used tool for gathering information about the gender identity and the self-perception of voice gender of transgender women. This assessment of the self-evaluation of voice gender informs professionals on how to help individuals who want their voice to match their gender identity (see [13] for a review of the effects of GAVT with transgender women).

The characteristics of perceived gender in the voice have been investigated for a long time, starting with the voices of cisgender people. The most common tool in the literature for investigating perceived binary gender is the visual analogue scale (VAS), a discrete or continuous scale on which participants indicate their perception along a spectrum (e.g. from ‘masculine’ to ‘feminine’ or ‘male’ to ‘female’) [14–17]. Alternatively, researchers have used categorization tasks (e.g. the two-alternative forced choice task; 2AFC), where participants must choose between different gender concepts [16,18–20]. Of course, the study of perceived binary gender has not been limited to cisgender individuals, and for several years, rating tools based on binary concepts—such as male/female or masculine/feminine—have also addressed the gender expressed by the voices of transgender individuals [21–25].

Understanding the perception and expression of gender in voices also involved investigating its acoustic representations. Over the years, studies have narrowed down the set of crucial acoustic features to a few key cues that strongly influence the perception of gender. The pitch, or fundamental frequency (F0), is the most robust predictor of perceived gender. Traditionally, higher F0 values are associated with more feminine voices, while lower F0 values signal masculinity [16,19,20,23,26,27]. Vocal tract resonances, reflected in the spacing and height of formant frequencies (especially F1–F4), also play a significant role, with higher formants linked to femininity and lower ones to masculinity [19,20,27,28]. Formant spacing was also related to the perception of gender, with bigger formant spacing driving higher perceived femininity [16,18,29,30]. Voice quality, particularly breathiness, has shown mixed influences on the perception of gender: auditory-perceptual studies suggest that breathiness increases perceptions of femininity [15,31], while others did not observe a significant contribution [17,32]. Despite the omnipresence of the above-mentioned features in the literature, other acoustic features have shown links with the perception of gender in the voice. For instance, dynamic changes in intonation and loudness patterns also contribute to gender perception. Thus, greater pitch variability and dynamic vocal intensity are generally perceived as more feminine [15,33,34]. See [35] for a comprehensive review of the acoustic representations of binary voice gender.

Research on gender-diverse voices has expanded significantly in recent years. Multiple studies have built upon tools originally designed for investigating binary gender while incorporating gender-diverse identity data. They typically investigated one or more of the following key relationships: (i) between gender expression and perceived gender, (ii) between gender expression and vocal cues, and (iii) between perceived gender and vocal cues. The present review aims to synthesize and evaluate current methods and findings in the study of gender diversity in the voice, with the goal to inform future research. Specifically, we seek to address the following questions:

[RQ1] what methods are used to assess gender identity, perceived gender and the acoustic properties of voices?

[RQ2] how are gender-diverse voice identities perceived?

[RQ3] what are the acoustic characteristics of gender-diverse voice identities?

[RQ4] what is the acoustic representation of perceived gender in the context of gender-diverse voices? and

[RQ5] does the identity of the listener influence the perception of gender-diverse voices?

Multiple studies on perceived and expressed gender diversity have been conducted in the context of evaluating the effect of GAVT methods [13] or GAHT. Although the efficacy and nature of gender-affirming procedures (GAVT and GAHT) are not the focus of this review, they are included and discussed because they constitute a substantial part of the investigations we reviewed and are relevant aspects of a speaker’s voice identity.

The article is structured as follows: after an outline of the methods used for the literature review, the results of the study are then presented in two sections. The first section presents the speech datasets and methods used to assess gender identity, perceived voice gender, and acoustic features across all studies. The second section presents findings regarding: (i) perceptions of gender-diverse voice identities, (ii) acoustic characteristics of gender-diverse voice identities, (iii) acoustic characteristics of perceived gender, and (iv) the influence of listener identity on perceived gender.

## 2. Methods

This article is an integrative review that aims to synthesize and critically analyse current approaches and findings in the study of vocal gender diversity in order to inform future research.

We used the databases PubMed and Web of Science to collect articles on the topic. We chose PubMed and Web of Science for their complementary coverage of published academic articles on gender diversity in the voice, with PubMed providing strong literature on health and mental well-being research and Web of Science offering multidisciplinary perspectives across the social sciences and humanities. The keywords used for the search were: (gender-diverse OR gender expansive OR transgender OR genderfluid OR genderqueer OR gender-neutral OR nonbinary OR non-binary OR gender-ambiguous) AND (voice OR speech) AND (perception OR production OR expression OR generation). We only included articles published in the English language in the past 5 years. We chose this time frame because methods in this field evolve rapidly, and more recent studies are most relevant for future practice. Thus, we collected a total of 331 articles on PubMed and 220 articles on Web of Science.

**Figure 1.**
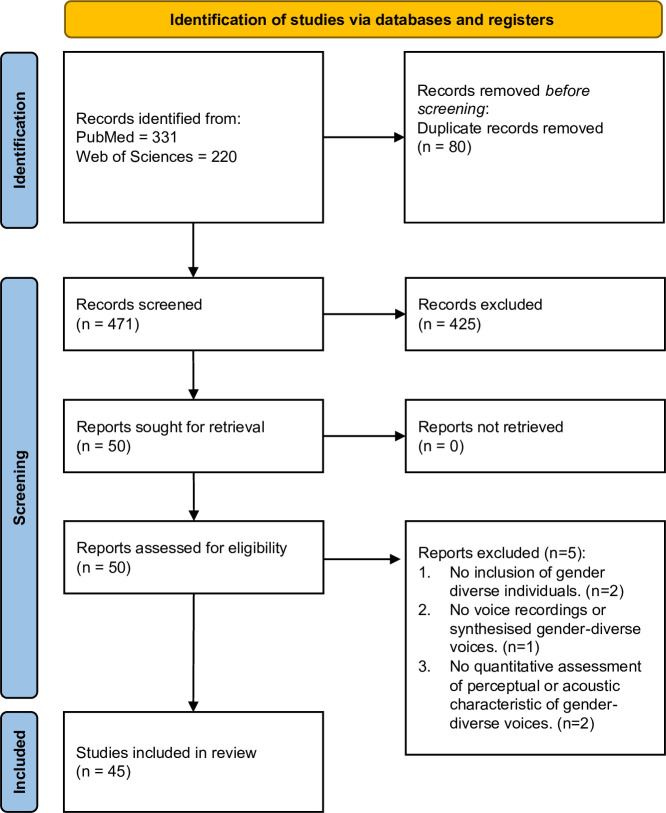
Flow chart of study selection.

After excluding duplicate articles between the two databases, we screened articles that focused on the human voice as an object of study by reading the titles and abstracts. We then read the methods sections of the selected articles and further screened papers based on the following inclusion criteria: (i) studies about gender non-conforming voices, (ii) studies including voice recordings of gender-diverse individuals or synthesized voices aiming to replicate gender-diverse voices, and (iii) studies including an acoustic analysis and/or a perceptual experiment. We excluded studies that investigated the perception or acoustics of gender-diverse voices without collecting quantitative data. The review process was led by the first author. Figure 1 presents the flowchart following PRISMA (Preferred Reporting Items for Systematic reviews and Meta-Analyses) guidelines for the selection of studies.

In total, we reviewed 45 studies published over the past 5 years, observing an increase in the number of publications: seven in 2020, six in 2021, five in 2022, nine in 2023, 15 in 2024 and three in January 2025 alone. Many of these articles (21) appeared in the *Journal of Voice*. All studies created or used speech datasets featuring either synthesized voices or voice recordings that included at least one gender-expansive voice identity (i.e. someone who is not a cisgender man or woman). In total, 34 studies incorporated a perceptual experiment using the speech dataset, and 38 conducted acoustic analyses. Although this review does not focus on speech therapy outcomes, we note that 18 studies from the present review specifically examined the effects of gender-affirming speech therapy methods. Table 1 shows the list of papers we included in the review, along with information on their content, i.e. whether they conducted a perceptual experiment and/or performed an acoustic analysis.

**Table 1.** Studies included in the review.

reference	journal	perceptual experiment	acoustic analysis
[36]	*Journal of Voice*	**yes**	no
[37]	*Journal of Voice*	no	**yes**
[38]	*Journal of Voice*	**yes**	**yes**
[39]	*Journal of Voice*	**yes**	**yes**
[40]	*Laryngoscope*	no	**yes**
[41]	*Journal of Voice*	no	**yes**
[42]	*Journal of Voice*	**yes**	**yes**
[43]	*International Journal of Transgender Health*	**yes**	**yes**
[44]	*American Journal of Speech-Language Pathology*	no	**yes**
[45]	*Journal of Voice*	no	**yes**
[46]	*Journal of Voice*	**yes**	**yes**
[47]	*Journal of Speech, Language, and Hearing Research*	**yes**	**yes**
[48]	*Journal of Voice*	**yes**	**yes**
[49]	*Journal of Voice*	**yes**	**yes**
[50]	*Journal of Voice*	**yes**	**yes**
[51]	*Journal of Voice*	**yes**	no
[52]	*Interspeech*	**yes**	**yes**
[53]	*Brain & Language*	**yes**	**yes**
[54]	*Seminars in Speech and Language*	**yes**	**yes**
[55]	*Interspeech*	no	**yes**
[56]	*Journal of Voice*	**yes**	**yes**
[57]	*Journal of Speech, Language, and Hearing Research*	**yes**	**yes**
[58]	*Journal of the Acoustic Society of America*	**yes**	**yes**
[59]	*Journal of Voice*	**yes**	**yes**
[60]	*American Journal of Speech-Language Pathology*	no	**yes**
[61]	*Journal of Voice*	**yes**	**yes**
[62]	*Journal of Voice*	no	**yes**
[63]	*International Journal of Transgender Health*	**yes**	no
[64]	*Journal of Voice*	**yes**	no
[65]	*Plos ONE*	**yes**	**yes**
[66]	*Journal of Voice*	**yes**	no
[67]	*Journal of Voice*	**yes**	**yes**
[68]	*Journal of Speech, Language, and Hearing Research*	no	**yes**
[69]	*Journal of Speech, Language, and Hearing Research*	**yes**	**yes**
[70]	*Journal of the Acoustic Society of America*	**yes**	**yes**
[71]	*Journal of the Acoustic Society of America*	**yes**	**yes**
[72]	*Journal of the Acoustic Society of America*	**yes**	**yes**
[73]	*Logopedics Phoniatrics Vocology*	no	**yes**
[74]	*Journal of the Acoustic Society of America*	**yes**	**yes**
[75]	*Journal of Speech, Language, and Hearing Research*	**yes**	no
[76]	*Journal of Voice*	**yes**	**yes**
[77]	*Folia Phoniatrica et Logopaedica*	**yes**	no
[78]	*Journal of Voice*	**yes**	**yes**
[79]	*Journal of Voice*	**yes**	**yes**
[80]	*Frontiers in Psychology*	no	**yes**

For all these studies, we extracted information based on the objectives of the present review.

Therefore, for all articles, we extracted:

- purpose of the study and study design;
- details on recording datasets (e.g. number and profile of speakers, speech material recorded, in-person/online);
- speakers’ gender-related demographics (e.g. gender identity, voice training, hormonal therapy);
- method to collect gender identity and self-evaluation of voice gender;
- main findings.

If there was a perceptual experiment, we additionally extracted:

- listeners’ demographics;
- perceptual dimension studied;
- method for assessing perceptual dimension.

If there was an acoustic analysis, we extracted:

- tool(s) used for acoustic analysis;
- acoustic features used;
- modelled dimensions (e.g. speaker gender identity, perceptual ratings).

## 3. Results

### 3.1. Experimental methods

In this section, we outline the speech datasets and methods used to assess gender identity, perceived voice gender, and acoustic features across all studies.

#### 3.1.1. Speech datasets

Most speech datasets were in English (29), with smaller numbers in Brazilian Portuguese (five), Dutch (six) and Swedish (four). Additionally, there were single instances of datasets in each of Hebrew, Spanish and French. While the majority of datasets consisted of speech recorded in-person (32 studies), some were recorded online (five studies), and others were sourced from pre-existing datasets (eight studies). Although most datasets contained samples of natural recorded voice, some studies also employed synthesized or acoustically manipulated speech based on natural voice samples [47,52–54]. The speech materials recorded or synthesized varied in type: the majority of recorded speech datasets featured both read speech (e.g. Harvard sentences, CAPE-V sentences, phonetically balanced texts) and spontaneous speech (e.g. descriptions of hobbies or images, dialogues). In total, 19 studies included recordings of sustained vowels (e.g. /a/, /i/, /o/) or vowels in carrier words (e.g. hVd, sVd). Additionally, four studies incorporated recordings of participants counting from 1 to 10, and three had participants performing vocal glides (i.e. pitch slide). Finally, two studies collected both phonated and whispered speech. Two of the datasets created are available online, either freely accessible [44] or upon request from the first author [55].

The speech datasets analysed in these studies varied significantly in terms of the number and diversity of gender identities represented. Traditionally, they included transgender and cisgender individuals who identified as men, women, masculine or feminine individuals, or as gender non-conforming (e.g. non-binary, genderfluid, agender). The number of explicitly distinguished gender identities per study ranged from 1 to 5. Transgender women were the most frequently represented group across the datasets (30 studies), followed by cisgender women (23 studies), cisgender men (23 studies), non-binary individuals (13 studies), transgender men (nine studies), transfeminine individuals (nine studies) and transmasculine individuals (seven studies). The size of the speech dataset used per study varied from one speaker to 251 speakers, with an average number of speakers of 43.

Table 2 provides a detailed overview of the speech datasets, including the number of speakers, their gender identities, speech material, language and whether speakers rated the perceived gender of their own voice.

**Table 2.** Overview of speech datasets: speaker gender identity, speech material and language. (Carrier words, hVd/sVd/bVd; N/A, not available. (°) denotes studies that used a validated scale (e.g. TWVQ, TSEQ, TVQ) to assess participants’ perception of their voice including the gender of their current voice, while (*) denotes studies in which speakers were asked to rate the gender of their own voice on a scale.)

reference	number of speakers	gender IDs	speech material	language
[36]	1	1 transmasculine individual	read speech	English
[37]	15	5 cisgender women 4 cisgender men 6 non-binary individuals	read speech spontaneous speech	English
[38]	1	1 transmasculine individual	read speech spontaneous speech vowels	English
[39]	115	32 cisgender women 21 cisgender men 31 transgender women 31 transgender men	counting vowels	Portuguese
[40] °	16	16 transgender women	read speech spontaneous speech	English
[41] °	4	4 non-binary/genderqueer individuals	read speech spontaneous speech vowels	English
[42] °	22	5 cisgender women 12 transfeminine individuals	read speech spontaneous speech vowels	English
[43] °	30	30 transgender women	read speech spontaneous speech vowels	Hebrew
[44]	20	cisgender men transgender men transmasculine individuals non-binary individuals	read speech	English
[45] °	45	45 transgender women	N/A	Spanish
[46] °	32	10 cisgender women 10 cisgender men 12 transwomen	carrier words	English
[47]	3	3 transmasculine individuals	read speech	English
[48]	17	15 transgender women 2 non-binary individuals	read speech spontaneous speech vowels	English
[49] °	40	10 cisgender women 10 cisgender men 20 transgender women	carrier words read speech vowels	English
[50] *	4	4 transgender women	read speech spontaneous speech	English
[51]	132	54 cisgender women 41 cisgender men 17 transgender women 12 transgender men 7 non-binary individuals	spontaneous speech	Swedish
[52]	N/A	synthesized voices	read speech	English
[53]	N/A	synthesized voices	read speech	English
[54]	N/A	synthesized voices	read speech	English
[55] *	13	13 gender-diverse individuals	read speech	English
[56]	30	10 cisgender women 10 cisgender men 10 transgender women	carrier words (phonated and whispered)	English
[57] °	24	8 cisgender women 8 cisgender men 8 transfeminine individuals	carrier words (phonated and whispered)	English
[58] °	35	17 cisgender women 11 cisgender men 7 transfeminine individuals	carrier words (phonated and whispered)	English
[59]	31	19 cisgender men 12 transgender women	carrier words	English
[60] °	30	30 transgender women	read speech spontaneous speech vowels	Dutch
[61] °	23	5 cisgender women 5 cisgender men 13 transgender women	read speech	Dutch
[62] °	30	30 transgender women	read speech spontaneous speech vowels	Dutch
[63] °	40	5 cisgender women 5 cisgender men 30 transgender women	read speech	Dutch
[64] °	40	5 cisgender women 5 cisgender men 30 transgender women	read speech	Dutch
[65] *	47	11 cisgender women 11 cisgender men 11 transgender women 7 transgender men 7 non-binary individuals	counting (1-10) read speech vocal gliding vowels	Portuguese
[66] *	47	11 cisgender women 11 cisgender men 11 transgender women 7 transgender men 7 non-binary individuals	counting (1-10) read speech	Portuguese
[67] °	40	20 cisgender women 20 transgender women	counting (1-10) read speech vowels	Portuguese
[68] °	62	60 transgender women 2 non-binary individuals	read speech	English
[69]	40	10 cisgender women 10 cisgender men 10 transfeminine individuals 10 transmasculine individuals	spontaneous speech	English
[70]	24	6 cisgender women 6 cisgender men 6 transfeminine individuals 6 transmasculine individuals	spontaneous speech	English
[71]	59	15 cisgender women 15 cisgender men 7 transgender women 7 transgender men 15 non-binary individuals	read speech	English
[72]	60	15 cisgender women 15 cisgender men 7 trangender women 12 non-binary individuals 1 agender individuals	read speech	English
[73] °	1	1 transgender women	spontaneous speech	French
[74]	132	54 cisgender women 41 cisgender men 37 gender-diverse individuals	spontaneous speech	Swedish
[75] °	114	22 cisgender women 18 cisgender men 74 transgender women	read speech	English Swedish
[76]	251	107 cisgender women 104 cisgender men 19 transgender women 10 transgender men 11 non-binary individuals	read speech	Dutch
[77]	34	22 transgender women 4 transfeminine individuals 8 non-binary transfeminine individuals	read speech spontaneous speech vocal gliding vowels	English
[78]	34	22 transgender women 4 transfeminine individuals 8 non-binary transfeminine individuals	read speech spontaneous speech vocal gliding vowels	English
[79]	114	22 cisgender women 18 cisgender men 74 transgender women	read speech spontaneous speech	English Swedish
[80]	61	31 cisgender women 30 transgender women	vowels	Portuguese

#### 3.1.2. Measuring gender identity and gender expression

Multiple studies have examined the impact of speakers’ characteristics on their voice. In many cases, researchers collected information on speakers’ gender identity, self-evaluation of voice gender, as well as additional factors relevant to voice gender, such as sex assigned at birth, history of gender transition and experiences with gender-affirming interventions.

##### 3.1.2.1. Gender identity of speakers and listeners

The method used to determine speakers’ or listeners’ gender identity was not always specified, but was probably self-reported at the time of recruitment. Some studies explicitly described their approach, with some allowing open-ended responses via a textbox [44,68] and others employing a multiple-choice questionnaire with a write-in option [72,74]. Alternatively, Hope *et al.* allowed listeners or speakers to rate their gender identity and gender expression using sliders for male, female and an ‘other’ gender identity, where the right endpoint of the scale represents something other than masculinity or femininity [52–55].

##### 3.1.2.2. Self-evaluation of voice gender

In total, 22 studies reported the self-evaluation of voice gender of speakers using a male–female scale or a masculine–feminine scale (table 2). Among them, 19 studies asked participants to also complete the TWVQ*,* TVQ^MtF^ (Transsexual Voice Questionnaire for Male-to-Female Transsexuals) or TSEQ (Transgender Self-Evaluation Questionnaire) [12]. These validated scales typically assess vocal satisfaction, gender congruence and the psychosocial impact of voice. For example, the TWVQ asks participants to rate statements like *‘I feel my voice does not reflect the “true me”’* or *‘I feel discriminated against because of my voice’*. They also ask participants to rate their own voice and ideal self-voice on a male–female scale.

##### 3.1.2.3. Speaker and listener characteristics related to voice gender

Additional information related to voice gender expression or perception was gathered from both speakers and listeners, either for participant screening or analysis. Speakers were sometimes asked about their sexuality, sex assigned at birth, and how they define their gender expression. Transgender and non-binary speakers could be asked to provide details about their transition, including their history of GAHT, GAVT, vocal cord or vocal fold surgery, and whether they had been diagnosed with gender dysphoria. Speakers might also report how long they had been presenting as their authentic self and in which context they do so in their daily life. Listeners could also be asked about their connection to the queer/gender-expansive community and their exposure to gender-diverse voices.

### 3.1.3. Measuring perceptual assessment of voice gender [RQ1]

Table 3 summarizes the different tasks and listener profiles used in studies on gender perception.

**Table 3.** Summary of participant demographics and listening tasks for studies that conducted a perceptual experiment. (The column ‘group*’* inform an investigation regarding the profile of listeners. In *italic* are tasks not related to gender perception. SLPs, speech and language pathologists; N/A, not available; *N*-AFC, *N*-alternative forced choice.)

reference	gender	group	task
[36]	20 cisgender 10 gender-diverse	10 cisgender younger 10 cisgender older 10 gender-diverse	male-female scale
[38]	8 cisgender	N/A	2AFC (-masculine/+masculine)
[39]	3 N/A	N/A	2AFC (male/female) *breathiness scale*
[42]	20 N/A	N/A	masculine-feminine scale
[43]	20 cisgender	N/A	masculine-feminine scale
[46]	24 N/A	N/A	male-female scale *5AFC word identification*
[47]	15 cisgender 5 gender-diverse	N/A	man-woman scale *naturalness scale*
[48]	10 cisgender 8 gender-diverse	10 cisgender 8 gender-diverse	masculine-feminine scale
[49]	20 N/A	N/A	3AFC (male/female/don’t know) masculine-feminine scale naturalness scale
[50]	10 N/A	N/A	masculine-feminine scale
[51]	77 cisgender 30 gender-diverse 14 SLPs	77 naïve 30 gender-diverse 14 SLPs	femininity scale masculinity scale
[52]	20 cisgender 20 gender-diverse	20 cisgender 20 gender-diverse	masculinity scale femininity scale other scale
[53]	28 cisgender 20 gender-diverse	28 cisgender 20 gender-diverse	masculinity scale femininity scale other scale
[54]	48 cisgender 31 gender-diverse	48 cisgender 31 gender-diverse	*2AFC word identification*
[56]	20 N/A	N/A	masculine-feminine scale
[57]	100 cisgender 5 gender-diverse	N/A	male-female scale masculine-feminine scale fem. female-masc. male scale femininity scale masculinity scale
[58]	2 N/A	N/A	*2AFC (correct/incorrect)*
[59]	26 N/A	N/A	male-female scale
[61]	22 cisgender	N/A	masculine-feminine scale
[63]	45 cisgender 16 gender-diverse 14 SLPs	N/A	masculine-feminine scale 3AFC (male/female/neutral)
[64]	31 cisgender 8 gender-diverse	N/A	masculine-feminine scale
[65]	101 cisgender 70 gender-diverse 65 SLPs	101 cisgender 70 gender-diverse 65 SLPs	masculine-feminine scale
[66]	101 cisgender 70 gender-diverse 65 SLPs	101 cisgender 70 gender-diverse 65 SLPs	masculine-feminine scale
[67]	3 N/A	N/A	masculine-feminine scale *emotion categorization*
[69]	52 N/A	N/A	2AFC(male/female) masculine-feminine scale *naturalness scale*
[70]	117 cisgender 6 gender-diverse	N/A	2AFC (male/female) masculine-feminine scale
[71]	30 cisgender	N/A	male-female scale masculine-feminine scale
[72]	42 cisgender	26 straight 16 queer	free classification on gender masculine-feminine scale male-female scale *free classification on similarity* *breathy-full scale* *clear-hoarse scale* *nasality scale* *likeness scale* *monotonous-animated scale* *slow rate-rapid rate scale* *unforced-effortful scale* *young-old scale*
[74]	77 cisgender 30 gender-diverse 14 SLPs	N/A	masculinity scale femininity scale
[75]	76 cisgender 3 gender-diverse	N/A	male-female scale
[76]	35 cisgender 6 gender-diverse	N/A	masculine-feminine scale
[77]	23 cisgender 35 gender-diverse	14 naïve 11 experts 34 gender-diverse	male-female scale masculine-feminine scale
[78]	23 cisgender 1 gender-diverse	14 naïve 11 experts	male-female scale masculine-feminine scale
[79]	76 cisgender 3 gender-diverse	N/A	male-female scale

There were various methods for asking listeners to rate the gender of a voice. Most commonly, voice gender was assessed by having participants listen to a voice sample and rate its gender using a VAS with different gradations (e.g. 5-point, 7-point, or 101-point scales). These scales typically rely on binary anchor terms, such as ‘male–female’ or ‘masculine–feminine,’ reflecting a traditional understanding of gender identity and expression. Some studies have challenged this approach, instead distinguishing between separate masculinity and femininity scales [51–54,57,74]. Houle *et al*. [57] further demonstrated that modifying anchor terms in this way yields different acoustic representations in the ratings, whereas Quinn *et al*. [77] found no differences between the ‘male–female’ and ‘masculine–feminine’ anchor terms. Investigating the perception of gender-ambiguous voices by gender-expansive speakers, Hope & Lilley also introduced an ‘other’ scale for a gender identity and gender expression different from masculine/male or feminine/female [52,53]. Other studies have used categorical response formats. These would usually be either 2AFC like male/female or masculine/feminine [38,39,70] or with additional non-binary options like ‘neutral’, ‘ambiguous’ [63], ‘genderfluid’, or ‘agender’ [53]. Taking a different approach, Merritt *et al*. [72] asked listeners to classify speakers on perceived gender identity to examine how salient gender is for the assessment of speaker identity.

A few studies have examined the impact of listeners’ demographics on voice gender perception [36,48,51–54,65,66,70]. The column ‘group*’* in table 3 reports the groups of participants in each of these studies.

Finally, in some cases, researchers investigated the impact of gender identity or perceived gender on other percepts like emotion, naturalness, voice quality, or word identification [39,46,47,49,67,69,72].

### 3.1.4. Measuring acoustic correlates of voice gender [RQ1]

Gender identity and perceived voice gender were also investigated from the perspective of acoustic representations, often using open-source analysis tools like Praat [81]. Here, we report the most often computed features.

Out of 38 studies that conducted acoustic analyses of voice samples, 35 measured the mean or median F0 of voice samples. Additional F0-related features were analysed, including F0 range (13 studies), variance (six studies) and jitter (three studies). Another group of features were primarily related to formants and vocal tract acoustics, with 22 studies extracting formants to predict perceived gender or to assess gender-based differences in speakers. Formants were mainly extracted as their average or median frequencies (22 studies), to compute vowel space densities (VSDs; six studies), or to compute the apparent vocal tract length (seven studies). Several studies also focused on spectral and noise-related features, including cepstral peak prominence (CPP; six studies), spectral moments (four studies), harmonic-to-noise ratio (HNR; seven studies) and glottal-to-noise excitation ratio (one study). In terms of intensity features, mean intensity was measured in seven studies and shimmer in six studies. Finally, prosody features were often investigated in terms of pitch contour or intonation shift (six studies) and speech rate (six studies).

See table 4 for the full report of extracted or manipulated acoustic features.

**Table 4.** List of extracted or manipulated acoustic features for each study. ((*) indicates studies that manipulated acoustic features of voice samples. s.d., standard deviation; CPPs, cepstral peak prominence; HNR, harmonic-to-noise ratio; VTI, voice turbulence index; SPI, soft phonation index; ABI, acoustical breathiness index; VSD, vowel space density; AVQI, acoustic vocal quality index; GNE, glottal noise excitation.)

reference	acoustic features
[37]	F0 (mean), CPPs, spectral slope
[38]	F0 (mean), formants (F1–F4)
[39]	F0 (mean), HNR, VTI, SPI, ABI
[40]	F0 (mean, range)
[41]	F0 (mean, range)
[42]	F0 (mean, range), formants (F1, F2)
[43]	F0 (mean, range), formants (F1–F3)
[44]	F0 (mean), formants, VSD, H1–H2, speech rate, spectral centroid, spectral variance
[45]	F0 (mean)
[46]	F0 (mean), formants (F2)
[47] *	F0 (mean, contour), formants (F1–F4)
[48]	F0 (mean)
[49]	F0 (mean, min, max, range), formants (F1–F3), HNR, shimmer, intensity, speech rate, intonation shift
[50]	F0 (mean, min, max, range)
[52] *	F0 (mean, contour), formants (F1, F2)
[53] *	F0 (mean, contour), formants (F1, F2)
[54] *	F0 (mean, contour), formants (F1, F2), spectral centroid
[55]	F0 (mean), formants (F1–F4), spectral centroid, peak frequency
[56]	F0 (mean), formants (F1–F3), vowel duration
[57]	F0 (mean), formants (F1–F3), vowel duration, CPPs
[58]	spectral centroid, spectral variance, spectral skewness, spectral kurtosis, duration
[59]	F0 (mean), formants (F1, F2)
[60]	F0 (mean), formants (F1–F5), VSD, intensity
[61]	F0 (mean), formants (F1–F5), VSD, speech rate
[62]	F0 (mean), formants (F1–F3), VSD, HNR
[65]	F0 (median, min, max, s.d., peak width), jitter, shimmer, spectral emphasis, intensity variance, HNR, H1–H2, CPPs, AVQI, ABI, speech rate, articulation rate
[67]	F0 (mean, range, contour), formants (F1–F3), GNE, intensity, jitter, shimmer, HNR, duration, speech range profile
[68]	F0 (mean), inflexion range
[69]	F0 (mean, range, s.d.), speech rate
[70] *	F0 (mean), formants, formant envelope change, intensity
[71]	F0 (mean), formants (F1–F3), VSD, CPPs
[72]	F0 (mean), formants (F1–F3), VSD, CPPs
[73]	F0 (mean)
[74]	F0 (mean), formants (F1–F4), VSD
[67]	see table 3 in [74]
[76]	F0 (mean, range, s.d.), intonation shift
[78]	F0 (mean, range), AVQI
[79]	F0 (mean, range, s.d.), formants (F1–F4), H1–H2, intensity
[80]	see table 1 in [80]

### 3.2. Findings

As discussed above, research has frequently explored how speakers’ gender identity and self-evaluation of voice gender relate to both externally perceived gender and acoustic features. Additionally, many studies have investigated the relationship between perceived gender and vocal cues. In this section, we summarize key findings on the interplay among the three dimensions of gender identity, perceived voice gender and acoustic cues.

#### 3.2.1. External perception of gender-diverse voices [RQ2]

In this section, we report key findings on the relationship between speakers’ gender identity and self-evaluation of voice gender with external perceptual evaluations of voice gender. As GAHT and GAVT were the focus of multiple studies and contribute to the voice identity of speakers, we also report how these interventions contribute to perceived gender.

In the case of speakers with diverse gender identities, we asked whether self-reported gender identity aligns with the perceived gender of their voice by other listeners. The voice of transgender women was reported to often be perceived either as significantly more male or more masculine than the voice of cisgender women and slightly less masculine than cisgender men voices [39,56,57,67]. Some studies observed that transgender voices are more ambiguous in terms of masculinity and femininity, in comparison to cisgender people. Merritt *et al*. reported that transgender voices were evaluated as less natural than the voice of cisgender individuals [69] and that transgender individuals tended to adopt cisgender intonation and articulation in line with their affirmed gender identity [70]. In a follow-up study, they showed that non-binary voices were rated intermediately between cisgender women and other identities including transgender and cisgender individuals [71]. Logically, multiple studies reported that the voice of cisgender speakers was rated as either masculine or feminine accordingly to their gender (e.g. [51,70]).

Similar to studies examining self-reported gender identity, self-evaluation of voice gender was compared with external evaluations. Thus, while Quinn *et al*. [77] found a correlation between self-ratings and external ratings, some studies reported discrepancies between self-perceived and externally-perceived voice gender. For example, in a study investigating the perception of the voice of transgender women, Diamant & Amir [43] demonstrated that the ratings of femininity by a population of cisgender listeners did not align with the scores derived from the TVQ^MtF^ completed by transgender speakers. Similarly, the self-assessment of voice gender using a masculinity–femininity slider significantly differed from the external evaluation of voice gender by speech and language pathologists (SLPs), cisgender individuals, and transgender individuals [66]. However, they observed that the external ratings from cisgender individuals and SLPs aligned with speakers’ ratings of their ideal voice. Finally, in another study investigating the perception of the voice of transgender women, while speakers tended to rate their own voice as more masculine than listeners did, listeners’ ratings and speakers’ ratings were significantly correlated [42].

GAHT and GAVT had a significant impact on the perception of gender from both the speakers themselves and external speakers. Two studies reported the evolution towards a perceptually more masculine voice with the use of GAHT for transmasculine individuals [36,47]. The use of puberty blockers by transfeminine individuals at later stage of adolescence had a more beneficial effect on self-voice evaluation (i.e. increased vocal satisfaction and perceived femininity) compared to earlier stage usage [45]. Regarding GAVT, many studies reported the benefits of various exercises of voice feminization on self-voice perception for transgender women and transfeminine individuals [40,61,64,68,73,75,78,79]. Similarly, two studies reported the efficiency of GAVT for transmasculine individuals [38] and non-binary individuals [41,68]. GAVT also had an impact on the externally-perceived gender of speakers, with many studies involving the voices of transgender women demonstrating higher femininity [50,59,61,63,75,78,79] after GAVT as perceived by cisgender and gender-expansive listeners. In the same way, Buckley *et al*. [38] found that GAVT enhanced the perceived masculinity of the voice of a transgender man.

#### 3.2.2. Acoustic characteristics of gender-diverse voice identities [RQ3]

The fundamental frequency was the main feature used for representing gender identity and self-voice gender perception. Thus, many studies found an effect of gender identity on F0-related features [37,39,44,45,56,59,65,67,71,80]. Most other acoustic analyses relied on spectral features, with studies observing an effect of self-reported gender identity or voice gender on formant frequencies, or VSD [44,59,67,71], and spectral moments [58]. Finally, other spectral and noise-related features were linked to gender identity or voice gender, including CPPs [37,65,71] and HNR [65,67].

Most of the time, acoustic analysis was used to reveal the distinction between the voices of transgender women and cisgender women [37,40,42,43,45,55,56,58,59,65,67,71,76,80]. However, five studies investigated the acoustic representations of the voices of transmasculine and non-binary individuals [44,55,65,71,76].

Table 5 provides a description of the acoustic features having a significant relationship with gender identity and/or self-voice gender perception.

**Table 5.** Significant effects of acoustic features on gender identities or scores of self-evaluated voice gender. (s.d., standard deviation; CPPs, cepstral peak prominence; HNR; harmonic-to-noise ratio; VTI, voice turbulence index; SPI, soft phonation index; ABI, acoustical breathiness index; VSD, vowel space density; GNE, glottal noise excitation.)

reference	dependent variables	gender IDs	acoustic features
[37]	gender identities	cisgender women cisgender men non-binary individuals	F0 (mean), spectral slope, CPPs
[39]	gender identities	cisgender women cisgender men transgender women transgender men	F0 (mean), ABI, SPI
[42]	male-female scale	cisgender women transfeminine individuals	F0 (mean), vocal intensity
[43]	TVQ^MtF^ scores	transgender women	F0 (mean, range)
[45]	TWVQ scores	transgender women	F0 (mean)
[55]	gender expression	gender-diverse individuals	formants
[56]	gender identities	cisgender women cisgender men transgender women	F0 (mean)
[58]	gender identities	cisgender women cisgender men transgender women	spectral centroid
[59]	gender identities	cisgender men transgender women	F0 (mean)
[65]	gender identities	cisgender women cisgender men transgender women transgender men non-binary individuals	F0 (mean, min, max, s.d., peakwidth), jitter, shimmer, HNR, CPPs, ABI
[67]	gender identities	cisgender women transgender women	F0 (mean, contour), formants (F1-F3), GNE, intensity, HNR
	TWVQ scores		F0 (mean)
[71]	gender identities	cisgender women cisgender men transgender women transgender men non-binary individuals	formants (F1-F3), VSD, CPPs
[80]	gender identities	cisgender women transgender women	F0 (mean, min, max, s.d.), jitter, shimmer, VTI

While some studies showed an effect of acoustics on self-voice perception ratings such as TVWQ scores or male–female/masculinity–femininity ratings [42,43,45,55,67], others found no significant relationship between acoustic features and self-evaluations of the gender of their voice [40,41,58].

Several studies have specifically examined the effects of GAVT on voice acoustics. Most focused on changes in F0 and formant frequencies, both of which showed significant shifts following intervention. In transgender women, GAVT was associated with increases in F0 [40,50,59–62,68,73,78,79] and formant frequencies [40,59–62,79]. Conversely, a transmasculine individual showed a decrease in F0 after training and an increase in (apparent) vocal tract length [38]. The only study to investigate voice training for non-binary individuals found no significant acoustic changes [41].

#### 3.2.3. Acoustic characteristics of perceived gender [RQ4]

Several studies focused their investigations on the relationship between perceived voice gender and voice cues. As the most investigated feature, F0 emerged as the strongest predictor of perceived gender, with higher F0 values correlating with increased femininity [42,43,47–49,56,57,59,71,74,79]. Formant frequencies, derived from the vowel content of speech, have also been shown to play a crucial role in gender perception across various voice types. In assessing voices from speakers with diverse gender identities, higher F1 values have been linked to more feminine voice perceptions [71], while variability in formant frequencies has also been found to influence perceived gender [74]. For transgender women’s voices, higher average formant frequencies were strong predictors of perceived femininity [43,79] with F2 interacting with F0 in shaping gender judgements [59]. Formants were found to be the most important feature in determining gender perception in a study using gender-ambiguous synthesized voices [52]. In whispered speech, where F0 is absent, F2 emerged as a key predictor of perceived gender [57]. However, findings on the role of formants were mixed, with one study reporting that they had had no effect on gender perception, and suggesting that other unaccounted-for factors also play a role [47].

The impact of pitch contour (pitch variation over time) on perceived gender appears to be relatively small [47,49,53,71]. Studies manipulating voices to make them more gender-ambiguous have found that intonation or pitch contour carries less weight compared to other features, such as articulatory cues [70] or vocal tract acoustics such as formant spacing [52]. However, while this holds true for perceived gender in cisgender and non-binary voices, moderate to strong correlations have been observed between different intonation profiles and femininity/masculinity ratings in the voices of transgender individuals [76].

Finally, while having a significant impact on perceived voice femininity, mixed results have been reported for intensity [42,74,79] and CPPs [57,74].

Table 6 reports the significant acoustic contributions to perceived gender.

**Table 6.** Significant effects of acoustic features on ratings of voice gender. (s.d., standard deviation; CPPs, cepstral peak prominence.)

reference	ratings	significant acoustic features
[42]	masculine–feminine	F0 (mean), intensity
[43]	masculine–feminine	F0 (mean, range), formants (F1)
[47]	man–woman	F0 (mean)
[48]	masculine–feminine	F0 (mean)
[49]	masculine–feminine	F0 (mean), formants, intensity
[52]	masculinity femininity other	formants
[53]	masculinity femininity other	formants
[56]	masculine–feminine	F0 (mean)
[57]	male–female masculine–feminine feminine female–masculine male femininity masculinity	phonated speech: F0 (mean), whispered speech: formants (F2), CPPs
[59]	male–female	F0 (mean), formants (F2)
[65]	masculine–feminine	see table 9 in [65]
[71]	masculine–feminine	F0 (mean), formants (F1)
[74]	masculinity femininity	F0 (mean), CPPs s.d., intensity, formants s.d.
[76]	masculine–feminine	intonation shift

#### 3.2.4. Impact of listener identities on perceived gender [RQ5]

Listener identities can influence the perception of voice gender. A series of studies by Hope & Lilley showed that gender-expansive listeners tend to have a more flexible and distinct representation of voice gender when using separate scales for masculinity, femininity, and an additional one for a gender expression distinct from masculinity and femininity [52,53]. Hope & Lilley [54] also reported that gender-expansive listeners and cisgender listeners perceive sibilants (/s/ or /ʃ/ sounds) differently. Similarly, transgender and non-binary listeners have demonstrated a distinct perception of voice gender compared to cisgender listeners [65] using masculine–feminine scales. On the other hand, some studies suggest that cisgender and gender-diverse listeners provide similar voice gender ratings [36,48,51].

Gender has been the primary identity factor examined; however, some studies investigated the perception of other demographic information about the listeners. While some studies found that experts such as SLPs rated voice gender differently from cisgender and gender-expansive listeners [51,65], others reported no effect of clinical expertise [77,78]. In a free classification task, Merritt *et al*. [72] found that straight and non-straight participants grouped voice genders similarly. Finally, exploring a different listener attribute, Brown *et al*. [36] reported that listener age did not influence perceptions of a transmasculine voice across stages of GAHT.

## 4. Discussion

In this review, we have presented recent methodological developments and findings in the research literature on gender-diverse voice identities. With 45 relevant studies published in the past 5 years, the number of investigations on gender-diverse voices is rapidly expanding. Although transgender women and transfeminine individuals have been the primary focus of much of this research, there is a growing inclusion of a wider range of gender identities, such as transgender male, transmasculine, non-binary, genderfluid and agender speakers. While many studies have built on binary-based methods to examine perceptual and acoustic aspects of gender diversity, a few others have developed new approaches to better reflect the complexity and fluidity of gender-diverse identities. Taken together, this body of work highlights both the progress being made and the limitations that remain in capturing the full spectrum of gender diversity in speech. Consequently, this discussion addresses four key areas: methods for investigating self-perception of voice gender among gender-diverse individuals; external perceptions of their voices; the impact of experimental design and participant characteristics on voice gender perception; and the acoustic features underlying gender-diverse voices and perceived voice gender.

In many cases, gender identity was self-reported using open-ended text responses, allowing a greater freedom for participants to describe their identity or categorical options allowing easier analyses. However, the collection methods were not always clearly reported, and standardization was often lacking. Recently, a growing body of research has focused on developing and standardizing questionnaires on gender identity and gender expression that could be used in this context [5,7,11,82–84]. By contrast, assessments of gender expression or the self-evaluation of voice gender in the reviewed papers tended to be more structured in a binary understanding of gender. Thus, influenced by standardized tools like the TWVQ, they often used male–female rating scales. As a result, while gender identity is often captured in flexible, open ways, (voice) gender expression is typically constrained to binary frameworks. This inconsistency of treatment between gender identity and voice gender may be a limitation when investigating non-binary and genderfluid voice identities with tools developed for binary categories. Inspired by the *Gender Unicorn* (https://transstudent.org/gender/), recent studies have addressed this issue by treating voice masculinity and femininity as separate dimensions and including ‘other’ or non-binary options in rating scales of gender expression [55]. The assessment of both gender identity and voice gender in this way, or by using other gender concepts (e.g. gender ambiguity, transgender, fluidity), offers a more inclusive and accurate framework for gender-diverse voice research.

Self-evaluation of one’s own voice is a valuable tool for understanding voice gender, but it has largely been assessed outside of experimental contexts. As mentioned above, most studies have used masculine–feminine rating scales or standardized questionnaires, such as the TWVQ, to evaluate the effectiveness of GAVT at single time points or longitudinally [62,64]. Thus, whereas speakers report how their voice *feels* in relation to their gender identity, listeners rate how a voice *sounds* in terms of perceived gender, untethered from self-identity. These are essentially two different concepts. As a result, this could be the reason why some studies observed discrepancies between how speakers evaluate their voice gender and how it is perceived by others [43,66]. Moreover, no study in this review has assessed self-perception through experimental tasks in which participants listen to their own voice in the same way external listeners do. To better understand the links between self- and other-perception of voice for gender non-conforming speakers and to evaluate self-voice satisfaction, future research should adopt experimental paradigms similar to those used in studies on self-voice perception (e.g. [85–87]).

Voices of gender-diverse speakers tended to be perceived as more ambiguous or misaligned with binary categories by listeners. Transgender women’s voices, for example, were typically rated as less feminine than cisgender women’s, but more feminine than cisgender men [39,56,67]. Similarly, perceived genders of non-binary and transmasculine voices often fell between binary categories [65,71]. Importantly, the perception of voice gender was reported to often be modulated by the use of GAHT [35,36] or the stage of GAVT undergone by speakers (e.g. [40,62,75,79]). Overall, in most cases, studies reported a mismatch between the speaker’s affirmed identity and the perception of their gender by listeners, with perceptions often falling into ambiguous categories or aligning with the speaker’s sex assigned at birth.

As noted above for the evaluation of the self-voice, perceived gender may be influenced by task design and listener identity or background. Most studies investigating perceived gender relied on VASs labelled ‘male–female’ or ‘masculine–feminine’ (e.g. [49,57]). As a binary presentation of gender, it may hinder the fluid understanding of gender expression by listeners. To counteract that, some have adopted separate masculinity and femininity scales [52–55,57,74] or included a third ‘other’ option to better reflect non-binary identities [52–54]. Future research should expand perceptual methods beyond binary formats to more accurately capture the diversity of voice gender perception. In addition to rating formats, listener identity also significantly influences how gender-diverse voices are perceived. We observed clear differences in how non-binary or gender non-conforming vocal identities are perceived, where listeners who identify as queer tend to demonstrate an understanding of queer voice identities that is distinct from that of cisgender listeners [52,53,55,65]. However, these findings are based on very few studies examining how listener identity or background influences the perception of gender non-conforming voices. Multiple studies have noted that the perception of gender identity in the voice interacts with other aspects of both speakers’ and listeners’ identities—such as age or cultural background [88,89]. This highlights the need to consider gender-diverse voice perception through an intersectional lens and to expand investigations on the conditions under which this perception of non-binary or gender non-conforming voices develops.

Similarly to research on binary voice gender, most studies including gender-diverse speakers have used acoustic analyses to examine both speakers’ gender identity and perceived gender. Unsurprisingly, F0 and formants (frequencies and spacing) emerge as key acoustic correlates in describing the voices of gender-diverse individuals. Among transgender women undergoing hormone therapy or voice training, these features typically shift away from cisgender men’s patterns and move closer to cisgender women’s norms [39,45,56,59]. However, when considering a wider range of acoustic parameters, some studies report substantial variability in acoustic profiles, particularly among transmasculine [44] and non-binary speakers [55]. This suggests that gender identity cannot be fully captured by conventional tools. In terms of perceived gender, fundamental frequency remains the strongest predictor of perceived gender, consistently linked to femininity or masculinity ratings. Formants also play a key role and may interact with pitch in shaping perception. Together, these findings confirm the central role of F0 and formants in gender perception, as it has previously been reported for cisgender speakers [35]. However, other acoustic cues—such as voice quality, intonation and articulatory dynamics—also influence how gender is perceived, though their weight varies across studies (see §3.2.3. for details about acoustic analyses results). While being less salient, these features nonetheless inform listeners' gender judgements. Houle *et al.* used whispered speech, removing pitch entirely, to demonstrate that formants alone could cue gender, emphasizing the hierarchical nature of acoustic gender cues [56,58]. Future research could take a similar approach, isolating specific features to better understand their individual impact on gender perception.

## 5. Conclusion and recommendations

In this review, we synthesized recent methods and findings in the study of gender diversity in voice. While many studies still draw on frameworks developed for binary gender, there is a growing shift towards approaches that aim to better represent the experiences and identities of transgender, non-binary, and other gender non-conforming individuals [RQ1]. Our findings revealed that compared to cisgender speakers, transgender and non-binary voices are often perceived as falling between binary categories or as misaligned with the speaker’s affirmed gender identity [RQ2]. Acoustically, gender identity, gender expression and perceived gender are vastly associated with fundamental frequency and formant structure [RQ3] [RQ4]. Finally, aspects of the listeners’ identity may influence how they perceive gender in the voice [RQ5].

These findings should be interpreted with caution for several reasons. First, many studies of this review continue to rely on binary rating tools and acoustic models that may not fully capture gender fluidity. Second, the literature remains skewed towards transfeminine speakers, often comparing their voices only to cisgender male and female norms, limiting our understanding of the broader spectrum of gender-diverse voices. Third, there is a notable lack of experimental data for assessing the self-perception of voice gender, which is essential to understanding how individuals experience their own voice in relation to their identity and external perceptions of gender.

Therefore, to reveal more comprehensive representations of gender-diverse voices, we recommend future research to:

(i) design more inclusive perceptual tasks that move beyond binary gender norms;
(ii) develop methods for assessing self-evaluations of voice gender that reflect the diversity of gender identities;
(iii) investigate how individuals perceive their own voice gender in experimental contexts; and
(iv) examine the impact of listeners’ demographics on perceived voice gender.

## Data Availability

This article has no additional data.
